# Editorial for the Special Issue: “Advances in Postoperative Pain Management and Chronic Postoperative Pain”

**DOI:** 10.3390/jcm11226667

**Published:** 2022-11-10

**Authors:** Marco Cascella

**Affiliations:** Division of Anesthesia and Pain Medicine, Istituto Nazionale dei Tumori, IRCCS Fondazione G. Pascale, 80100 Napoli, Italy; m.cascella@istitutotumori.na.it

Acute and chronic pain are two completely distinct universes. The clinical aspects underlying their physiopathology, epidemiology, and therapeutic problems are different. Acute pain is generally caused by a definite illness or trauma, and is usually limited to the period of time required to repair the damage. By contrast, chronic pain is not just a symptom and becomes a disease itself, affecting different aspects of the patient′s health-related quality of life [1]. In this context, the biological injury is no longer the main actor. Chronic pain is not a linear experience directly induced by sensory inputs that are evoked by the stimulation of nociceptors (“nociception”) [2]. It is a multidimensional experience induced by the activation of a diffuse brain network (pain matrix) and involving a widely distributed neural network. A complex series of biopsychosocial phenomena paint clinical pictures that often highlight a cause (secondary chronic pain), although some subtypes of chronic pain are not directly related to a disease (primary chronic pain) [1]. Consequently, the management of this leading source of suffering can be very challenging [3].

Pain occurring after surgery is called postoperative pain (POP). This type of acute pain is usually predictable and is characterized by a high intensity and short duration (usually 2 to 5 days). The clinical aspects of POP may vary from patient to patient and for the same individual over time. They depend on the pre-existing pathology and anatomy as well as on the type and invasiveness of the surgical approach. Distinct variables such as psychological factors, as well as cultural, religious, and socioeconomic aspects, and other components may impact the clinical features of the pain.

Clinically, POP involves various troublesome sensory and emotional experiences that may or may not be combined with autonomic and behavioral changes. When excessive and prolonged, these complicated humoral responses, which are originally aimed at maintaining homeostasis, can provoke organic, psychological, and behavioral changes such as anxiety, insomnia, and depression. If not accurately managed, POP can develop into a composite chronic pain problem [4]. In this context, chronic postsurgical pain (CPSP) is characterized by painful symptoms at the site of the surgical wound or in related areas. This pain lasts for more than 3 months and is not due to surgical complications [1]. Although the magnitude of the phenomenon varies according to the type of surgery, approximately one patient in four suffers from mildly severe CPSP, and one in six suffers from very severe symptoms [5].

CPSP is associated with significant discomfort, distress, and disability. Given the clinical and social impacts of CPSP, its prevention is of paramount importance. Regional anesthesia techniques can represent a favorable approach to addressing this issue [6]. In a prospective cohort study, Wiech et al. [7] assessed the effects of bilateral erector spinae plane block (ESPB) on the severity and incidence of CPSP in patients undergoing coronary artery bypass grafting via sternotomy. They administered 0.375% ropivacaine under ultrasound guidance before the induction of general anesthesia and proved that, compared with that in the control group (*n* = 24), the CPSP intensity was lower in patients treated with the block (*n* = 27). The result was significant at 1, 3, and 6 months after surgery (*p* < 0.001). Significant differences in favor of the ESPB group were also reported for the opioid intake, mechanical ventilation time, and hospital length of stay.

Regional anesthesia techniques have also been investigated for evaluating the short-term effects of preventive analgesic strategies on POP. In a single-blinded, randomized controlled trial, Kim et al. [8] evaluated the analgesic efficacy of ESPB combined with intercostal nerve block (ICNB). In particular, they compared this combination with a strategy focused on the association of thoracic paravertebral block (PVB) and ICNB in patients (*n* = 52) who underwent video-assisted thoracic surgery (VATS). The results showed that ESPB was not inferior to PVB when combined with ICNB for addressing pain at 24 and 48 h after the operation.

Thoracic ESPB is a simple and secure procedure. It can provide effective postoperative analgesia [9] and reduce the opioid consumption and length of stay after surgery [10]. Hochberg et al. [11] conducted a retrospective analysis to evaluate the efficacy of ESPB (10 mL of 1% lidocaine plus 10 mg of dexamethasone for a unilateral technique; 15–20 mL of 1% lidocaine and the same dose of the synthetic glucocorticoid for a bilateral approach) in patients suffering from pain related to chronic thoracic cancer (*n* = 44) and non-cancer-related pain (*n* = 66). Their results showed a mean reduction in pain scores (NRSs) of 2.4 points (*p* > 0.001) compared to the pre-procedure values. Prospective trials should be conducted to better define the operative modalities in different clinical settings.

Divergences between women and men in pain concern both sex and gender, where the word “sex” applies to human anatomy and physiology, and the word “gender” is related to psychosocial relationships [12]. Raak et al. [13] conducted a secondary analysis of a randomized controlled clinical trial for studying gender differences after lumbar spinal sequestrectomy due to degenerative diseases of the lumbar spine. The outcomes included the pain intensity (VAS), affective and sensory pain perception, and morphine equivalent doses of opioid painkillers used. The authors found no significant differences in any of the outcomes between men (*n* = 46) and women (*n* = 42). Since previous investigations found worse pain, disability, and quality of life in women than in men [14], further studies are warranted to clarify the gender differences in this clinical setting [15].

Several innovative and interesting mathematical approaches can be applied to the study of POP. For example, pain can be viewed as a trajectory rather than as one or more simple point estimates of its degree [16]. In a prospective monocentric cohort study, Dorges et al. [17] studied the pain trajectories in patients who underwent posterolateral (*n* = 92) or axillary thoracotomies (*n* = 89). They recognized four trajectories of postoperative pain, including a “worst” trajectory (30%) with constantly high pain; a trajectory with constantly low pain (32.6%); another trajectory with a steep decrease in pain (22.7%); and, finally, a trajectory featuring a steep increase (15%). Notably, the risk factors for chronic pain were the occurrence of preoperative pain (OR = 6.94; CI 95% (1.54–31.27)) and scar length (OR = 1.20 (1.05–1.38)). On the contrary, ASA class III seemed to be a protective factor for the worst pain group (OR = 0.02 (0.001–0.52)).

As highlighted in other articles collected in this Special Issue, regional anesthesia techniques are an interesting field of study. Researchers are seeking innovative approaches that are easy and safe. In a case report, Petrucci et al. [18] described an adjusted technique for ultrasound-guided thoracic PVB via the thoracic intervertebral foramen in a patient who underwent emergent laparotomy due to a small intestinal volvulus. They used two continuous catheters for a bilateral extended block (levobupivacaine 0.25% at a rate of 5–8 mL/h) and conducted clinical observations and virtual dissections to demonstrate the effective spread of the injection. The “Petrucci’s block” is a fascinating perspective, but a cadaver study is necessary to refine the technique and proceed with its clinical validation.

In pregnant women, cerebrospinal fluid outflow after spinal anesthesia for a cesarean section can cause headaches, vomiting, and nausea. Since ketamine increases intracranial pressure, it could prevent postpuncture symptoms due to perioperative hypocranial pressure. Liang and collaborators [19] conducted a systematic review and meta-analysis on the topic. The search strategy yielded 12 randomized trials (*n* = 2099), and, interestingly, there was no significant association between intravenous ketamine and the improvement of symptoms due to hypocranial pressure.

Chronic cancer pain is a subset of chronic secondary pain produced by a primary cancer itself, its metastases, or its therapy [1]. Its management requires a complex and multimodal approach. Telemedicine-based strategies can be adopted for this aim [20]. Nevertheless, there is a need for establishing the correct model of care by combining remote consultations and in-person visits for emergencies or for diagnostic or clinical aims. Predictive models are a meaningful opportunity in medicine, and machine learning (ML) approaches can probably be used for designing careful telemedicine processes. In a study conducted at the Istituto Tumori Fondazione Pascale (Italy), the authors implemented different ML models including random forest, gradient boosting machine, a single layer artificial neural network, and the LASSO–RIDGE algorithm to define the variable(s) that can influence the number of remote consultations. The ML-based simulations demonstrated that this parameter can be influenced by selected variables, mostly the patient’s age, the cancer type, and the occurrence of bone metastases [21].

Multimodal analgesia is fundamental for acute and chronic pain management. Nefopam is a centrally acting, non-opioid, non-steroidal analgesic drug. Several studies have demonstrated its efficacy for multimodal analgesia [22]. In a randomized, single-blinded, controlled trial reported in this Special Issue, the authors evaluated the analgesic efficacy of the intraoperative administration of nefopam (20 mg after induction and 15 min before the end of surgery) [23]. The patients of the nefopam group (*n* = 50) and control (*n* = 49) underwent VATS for lung cancer. The results indicated that the pain intensity at 72 h after the operation and after 3 months (chronic pain) and the total opioid consumption did not vary between the groups. Since nefopam has not been assessed in this setting, the results have important value in this research field.

In conclusion, the articles in this Special Issue should be helpful for identifying gaps in the prevention, diagnosis, and management of acute and chronic pain issues.
